# Effects of Low-Molecular-Weight Fucoidan and High Stability Fucoxanthin on Glucose Homeostasis, Lipid Metabolism, and Liver Function in a Mouse Model of Type II Diabetes

**DOI:** 10.3390/md15040113

**Published:** 2017-04-07

**Authors:** Hong-Ting Victor Lin, Yu-Chi Tsou, Yu-Ting Chen, Wen-Jung Lu, Pai-An Hwang

**Affiliations:** 1 Department of Food Science, National Taiwan Ocean University, No. 2, Pei-Ning Road, Keelung 202, Taiwan; HL358@ntou.edu.tw (H.-T.V.L.); miss350100@gmail.com (W.-J.L.); 2 Center of Excellence for the Oceans, National Taiwan Ocean University, No. 2, Pei-Ning Road, Keelung 202, Taiwan; 3 Department of Bioscience and Biotechnology, National Taiwan Ocean University, No. 2, Pei-Ning Road, Keelung 202, Taiwan; D97360002@mail.ntou.edu.tw (Y.-C.T.); m3kitty41221@gmail.com (Y.-T.C.)

**Keywords:** type II diabetes, db/db mice, brown algae, fucoidan, fucoxanthin, glucose homeostasis, lipid metabolism, live function

## Abstract

The combined effects of low-molecular-weight fucoidan (LMF) and fucoxanthin (Fx) in terms of antihyperglycemic, antihyperlipidemic, and hepatoprotective activities were investigated in a mouse model of type II diabetes. The intake of LMF, Fx, and LMF + Fx lowered the blood sugar and fasting blood sugar levels, and increased serum adiponectin levels. The significant decrease in urinary sugar was only observed in LMF + Fx supplementation. LMF and Fx had ameliorating effects on the hepatic tissue of db/db mice by increasing hepatic glycogen and antioxidative enzymes, and LMF was more effective than Fx at improving hepatic glucose metabolism. As for glucose and lipid metabolism in the adipose tissue, the expression of insulin receptor substrate (IRS)-1, glucose transporter (GLUT), peroxisome proliferator-activated receptor gamma (PPARγ), and uncoupling protein (UCP)-1 mRNAs in the adipose tissue of diabetic mice was significantly upregulated by Fx and LMF + Fx, and levels of inflammatory adipocytokines, such as adiponectin, tumor necrosis factor-α (TNF-α), and interleukin-6 (IL-6), were significantly modulated only by LMF + Fx supplementation. The efficacy of LMF + Fx supplementation on the decrease in urinary sugar and on glucose and lipid metabolism in the white adipose tissue of db/db mice was better than that of Fx or LMF alone, indicating the occurrence of a synergistic effect of LMF and Fx.

## 1. Introduction

Several medical conditions can cause hyperglycemia, of which diabetes is the most common by far. In diabetes, blood sugar levels increase because of insufficient insulin in the body or because body cells cannot efficiently unitize insulin. Hyperglycemia is a condition in which an excessive amount of glucose circulates in the blood, which plays an important role in the development of type II diabetes (non-insulin-dependent diabetes). When the pancreatic β-cells fail to secrete sufficient insulin to overcome insulin resistance, hyperglycemia becomes evident [1]. Diabetes is also associated with diseases, such as nephropathy [2], retinopathy [3], chronic renal failure [4], and cardiomyocyte apoptosis [5]. Therefore, an effective control of the blood sugar level is the key to prevent or modulate diabetic complications.

More than ten classes of drugs are currently available for diabetes treatment, and they differ in terms of safety and pathophysiological effects. However, in addition to inadequate efficacy and durability, some of these agents have disadvantages, such as causing hypoglycemia, nausea, weight gain, lactic acidosis, pancreatitis, liver disease, gastrointestinal intolerance, and heart failure [6,7]. Interest in the therapeutic use of natural products for diabetes treatment has been growing. Previous studies on the isolation and production of hypoglycemic extracts mainly focused on plants, such as garlic [8], ginger, fenugreek seed [9], and guava [10]. Other studies investigated hypoglycemic extracts from microorganisms, such as brewer’s yeast [11], probiotics [12], *Monascus* [13], and *Ganoderma* [14].

Brown seaweeds are well known for their abundant bioactive substances, such as the sulfated polysaccharide fucoidan [15], the carotenoid fucoxanthin [16], phlorotannin [17], and polyphenolic compounds [18]. Fucoidan has been reported to exhibit antioxidant [19], anticoagulant [19], antiinflammatory [20,21], hypolipidemic [22], and anticancer activities [23]. In addition, previous studies demonstrated that fucoidan can improve osteogenic differentiation, and low-molecular-weight fucoidan was found to be more potent than its high-molecular-weight equivalent [24]. Jeong et al. [25] also indicated that low-molecular-weight fucoidan can improve endoplasmic reticulum stress-reduced insulin sensitivity through adenosine monophosphate (AMP)-activated protein kinase activation in L6 myotubes, and it can restore lipid homeostasis in a mouse model of type II diabetes. Fucoxanthin, a carotenoid present in the chloroplasts of brown seaweeds, also exhibits strong antiobesity and antidiabetic effects on diet-induced obesity conditions in vivo [26]. Our previous study demonstrated that fucoxanthin-rich extract from the brown seaweed *Sargassum hemiphyllum* can inhibit α-amylase, α-glucosidase, sucrase, and maltase activities and stimulate insulin secretion in vitro [27].

The role of adipose tissue in the secretion of the biologically active mediators adipokines, such as adiponectin, leptin, and tumor necrosis factor (TNF)-α, has recently been recognized. These adipokines are reported to alter insulin sensitivity and glucose metabolism in the liver, adipose, and muscle tissues [28]. The liver plays a crucial role in carbohydrate metabolism because it is responsible for balancing blood glucose levels via glycogenesis and glycogenolysis [29]. In the presence of liver disease, metabolic glucose homeostasis is impaired because of disorders, such as insulin resistance, and the etiology of liver disease is important in the incidence of diabetes [30]. Thus, diabetes is currently the most common cause of liver disease, and liver disease is an important cause of death in people with type II diabetes [31].

Therefore, in this study, we aimed to further investigate the effects of low-molecular-weight fucoidan (LMF), high-stability fucoxanthin (Fx), and the combination of LMF and Fx on glucose homeostasis, lipid metabolism, and liver function in a mouse model of type II diabetes (leptin receptor-deficient db/db mice) [32]. The liver condition and the mechanism of insulin resistance in the adipose tissue were observed by determining the expression of glucose metabolism-related genes. To our knowledge, no reports describing the combined effects of fucoidan and fucoxanthin in terms of antihyperglycemic, antihyperlipidemic, and hepatoprotective activities in a mouse model of type II diabetes have been published.

## 2. Results and Discussion

### 2.1. Effect on the Body Weight and Food Intake

Type II diabetic db/db mice are non-insulin-dependent diabetes mellitus model animals that show symptoms similar to those of diabetes in obese humans. These mice exhibit overeating and obesity caused by an abnormality in the leptin receptor as well as increases in weight and blood sugar levels at 3–4 weeks after birth. At 9 weeks of age, these mice exhibit high body weight and blood sugar levels in the stationary stage, according to the manufacturer’s manual (Institute for Animal Reproduction, Kasumigaura, Ibaraki, Japan).

**Figure 1:**
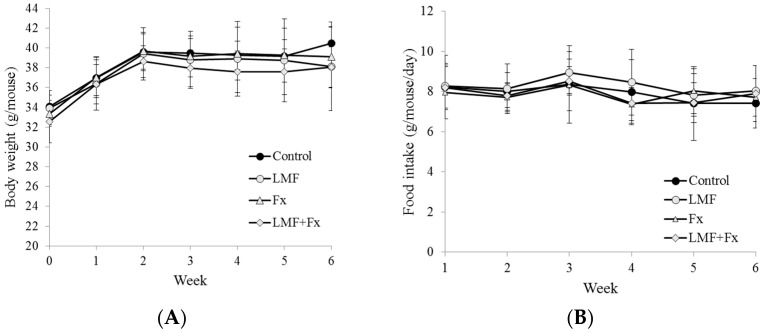
Effects of low-molecular-weight fucoidan (LMF), high-stability fucoxanthin (Fx), and LMF + Fx on (**A**) body weight and (**B**) food intake of db/db mice for 6 weeks. Body weights were monitored weekly. Food intake was measured weekly, and the values are the average weight of food consumed per mouse per day. Values were expressed as mean ± standard deviation (SD) (*n* = 8).

Diabetes treatment can occasionally cause increased weight gain; for example, thiazolidinediones for treating diabetes can induce adipogenesis in cell culture models and can increase weight gain in rodents and humans [33]. As shown in Figure 1, the intake of LMF, Fx, and LMF + Fx (300 mg/kg of the body weight) did not increase the body weight of the mice, and the intake of LMF + Fx slightly decreased it. The food intake is shown in Figure 1B. The food intake in all groups of mice were in the range of 7.4 to 8.9 g per day for 6 weeks, and the total food intake (6 weeks) for each group of the mice was 331.9 ± 62.9 g (db/db), 348.0 ± 51.5 g (db/db-LMF), 329.8 ± 43.2 g (db/db-Fx), and 331.2 ± 40.5 g (db/db-LMF + Fx), individually. Both total and daily food intake were not statistically different among all groups of the mice. It was also previously reported that the feeding of db/db mice on *Ecklonia cava* for 6 weeks had no effects on weight gain [34]. In addition, Lee, et al. [34] indicated that the dieckol rich extract of *E. cava* exerted an anti-diabetic effect in db/db mice by improving the glucose and lipid metabolism and antioxidant enzymes. Jeong et al. [25] also reported a decrease in the white adipose tissue when db/db mice were treated with a high dose of low-molecular-weight fucoidan (500 mg/kg of the body weight) from *Undaria pinnatifida*. Therefore, it was suggested that the natural compounds fucoidan and fucoxanthin from brown seaweeds do not increase the body weight of db/db mice but do inhibit weight gain at higher dosages. Therefore, for the rest of the study, we evaluated the effects of LMF, Fx, and the combination of LMF and Fx on glucose homeostasis, lipid metabolism, and liver function.

### 2.2. Reduction in Insulin Resistance

Hyperglycemia can cause major health complications in people with diabetes. Metformin and sulfonylureas are the most widely used oral therapies for type II diabetes, they lower glucose levels and reduce complication rates. However, their ability to maintain glycemic control can weaken, and they are also associated with weight gain and hypoglycemia [35].

**Figure 2:**
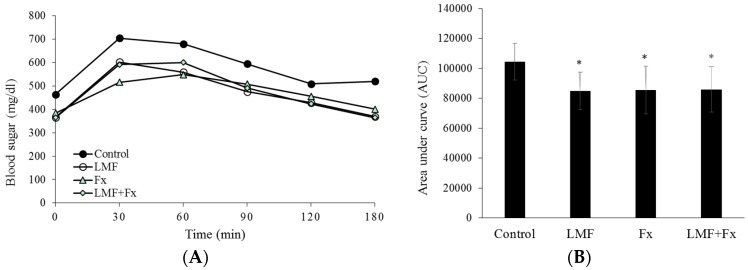
Effects of LMF, Fx, and LMF + Fx on (**A**) intraperitoneal glucose tolerance analysis and (**B**) area under the curve (AUC) of db/db mice for 6 weeks. After a 12-h fast, the mice were intraperitoneally injected with glucose (1 g/kg body weight). AUC was calculated by trapezoidal rule and the values represent the AUC from 0 to 180 min after glucose loading. Values were expressed as mean ± SD (*n* = 8). * *p* < 0.05 when compared with the db/db group.

As shown in Figure 2A, after 12 h of fasting, the db/db mice treated with LMF, Fx, and LMF + Fx exhibited a lower blood sugar level of 365–384 mg/mL than that in control db/db mice (464 mg/mL). After administering a glucose solution, blood sugar levels in all groups peaked at 30 min, among which the control db/db mice exhibited the highest blood sugar levels. Figure 2B shows the significant effects of LMF, Fx, and LMF + Fx in terms of reducing insulin resistance. Area under the curves (AUCs) of the db/db mice supplemented with LMF, Fx, and LMF + Fx were significantly decreased by 18.66%, 18.15%, and 17.71%, respectively, compared with those of the control db/db mice. We further investigated the insulin sensitivity of the db/db mice treated with LMF, Fx, and LMF + Fx. As shown in Table 1 these mice showed reduced fasting blood sugar levels, although decreases in serum insulin levels were not statistically significant. Serum adiponectin levels are closely related to systemic insulin sensitivity, and reduced serum adiponectin levels are considered a characteristic of type II diabetes [36].

Adiponectin administration has been shown to be accompanied by lower plasma glucose levels as well as increased insulin sensitivity [37]. Thus, we further investigated serum adiponectin levels and found that the db/db mice supplemented with LMF, Fx, and LMF + Fx had significantly higher serum adiponectin levels and marginally decreased serum insulin levels compared with those in the control db/db mice. Homeostatic model assessment (HOMA)-IR is an index of insulin resistance, and a lower HOMA-IR indicates higher insulin sensitivity. HOMA-β is an index of β-cell function, and a higher HOMA-β indicates greater insulin secretion. Our results demonstrated a higher HOMA-IR in the db/db mice treated with LMF, Fx, and LMF + Fx than that found in the control mice, whereas the increase in HOMA-β was marginal (Table 1). Based on the improvement in glucose tolerance and the decrease in serum insulin levels, it was suggested that LMF, Fx, and LMF + Fx could enhance insulin sensitivity. This was consistent with the results of the previous studies by Jeong et al. [25] and Kim et al. [38], who reported that blood sugar levels in type II diabetic mice were significantly decreased by fucoidan and that fucoxanthin-rich *Undaria pinnatifida* lipid extract reduced blood sugar levels in mice with obesity induced by a high-fat diet [26]. Jeong, et al. [25] indicated that the blood adiponectin levels in db/db mice supplemented with low-molecular weight fucoidan were increased by 50%, and the combination supplementation of LMF + Fx in this study increased the adponectin levels by more than 100% (Table 1).

### 2.3. Reduction in Urinary Glucose Level

**Figure 3:**
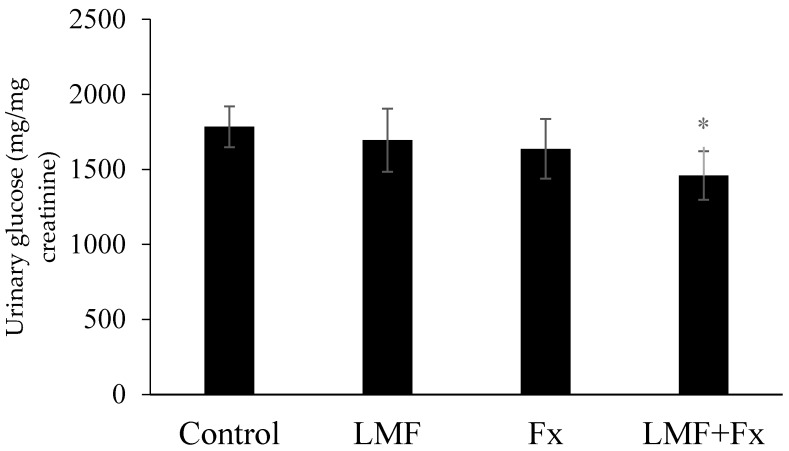
Effects of LMF, Fx, and LMF + Fx on urinary glucose of db/db mice for 6 weeks. Values were expressed as mean ± SD (*n* = 8). * *p* < 0.05 when compared with the db/db group.

Glycosuria involves glucose excretion into the urine, which is almost always caused by elevated blood sugar levels, mostly because of untreated diabetes [39]. To our knowledge, the effects of fucoidan and fucoxanthin on urinary glucose levels in a diabetic mouse model have not been determined. As shown in Figure 3, the supplementation of LMF and Fx separately marginally decreased the urinary glucose level in db/db mice, whereas the combined treatment using LMF + Fx lowered it significantly, indicating the synergistic effects of this combination treatment. Ben Rebah, et al. [40] reported that crude extract from *Caulerpa prolifera* markedly reduced both dog gastric and human pancreatic lipase activities, but the fractionations of the crude extract reduced the inhibitory rate. It was suggested that bioactivities may be caused by synergistic action of several compounds in the extract, and LMF + Fx showed a stronger effect on urinary glucose levels than LMF or Fx alone. Based on these physiological parameters, we hypothesized that LMF, Fx, and LMF + Fx can prevent and treat diabetes by reducing the blood sugar level and renal burden. To determine the possible synergistic effects of LMF and Fx, we further investigated the effects of LMF, Fx, and LMF + Fx on the liver and the mechanism of insulin resistance in adipose tissues of db/db mice.

### 2.4. Effects on Hepatic Glycogen Levels and Hepatic Functions

Liver glycogen metabolism plays an important role in glucose homeostasis. Alanine aminotransferase (ALT) and aspartate aminotransferase (AST) are enzymes normally found in liver cells, but they leak out of these cells into the blood when liver cells are damaged, thereby making them markers of liver function. AST catalyzes the interconversion of aspartic and α-ketoglutaric acids to oxaloacetic and glutamic acids, and ALT catalyzes the interconversion of alanine and α-ketoglutaric acid to pyruvic and glutamic acids. Thus, these enzymes act as a bridge between protein and carbohydrate metabolisms, resulting in the inclusion of keto acids in the tricarboxylic acid cycle [41]. In diabetes, less glycogen is stored in the liver, which contributes to postprandial hyperglycemia [42]. As a result, AST and ALT levels are elevated to produce alternative glucose precursors to compensate [43]. Our data showed that hepatic glycogen levels were significantly higher in db/db mice supplemented with LMF and LMF + Fx than in the control mice (Table 2). Unsurprisingly, reduced AST and ALT levels were observed in db/db mice supplemented with LMF and LMF + Fx. Although not statistically significant, the intake of Fx caused a marginal increase in hepatic glycogen levels and a decrease in ALT and AST levels. Heeba and Morsy [44] also reported that rats fed with high-fat diet would increase their serum AST and ALT levels, but these increases were suppressed by the treatment of fucoidan. Further, fucoidan also increased hepatic reduced antioxidant to ameliorate insulin resistance. According to our data, it was suggested that LMF ameliorated insulin resistance by elevating liver oxidative activities (superoxide dismutase (SOD) and catalase (CAT)) and enhancing liver function (ALT and AST), and may have the potential to be used for treating diabetes.

Nonalcoholic fatty liver disease (NAFLD) is a condition in which excessive fat accumulates in the liver of patients who drink no or little alcohol [45]; NAFLD development is closely associated with obesity and type II diabetes mellitus [46]. Oxidative stress is the crucial pathophysiological mechanism in NAFLD, causing NAFLD to progress to liver cirrhosis [45], and this oxidative stress can be detected by measuring the activity of antioxidative enzymes, such as superoxide dismutase (SOD) and catalase (CAT) [47]. As shown in Table 3, in db/db mice supplemented with LMF, Fx, and LMF + Fx, there were increases in the levels of the antioxidative enzymes SOD and CAT in the liver, indicating that LMF and Fx supplementation had antioxidant defensive actions against diabetes. Intriguingly, no significant synergistic effect was observed on antioxidative enzyme levels when db/db mice were treated with the combination of LMF and Fx.

Our results suggested that LMF and Fx have ameliorating effects on the hepatic tissue of db/db mice by increasing hepatic glycogen and antioxidative enzyme levels and that LMF is more effective than Fx at improving hepatic glucose metabolism. Hu et al. [48] reported that fucoidan improves glucose metabolism via increases in hexokinase and pyruvate kinase activities and reductions in glycogen phosphorylase and glucose-6-phosphatase activities. It is essential to determine the mechanisms underlying the synergistic effect of LMF + Fx on urinary glucose levels and insulin sensitivity, therefore, glucose and lipid metabolism in the adipose tissue were investigated.

### 2.5. Effect on mRNA Expression Related to Glucose and Lipid Metabolism in the Adipose Tissue

**Figure 4:**
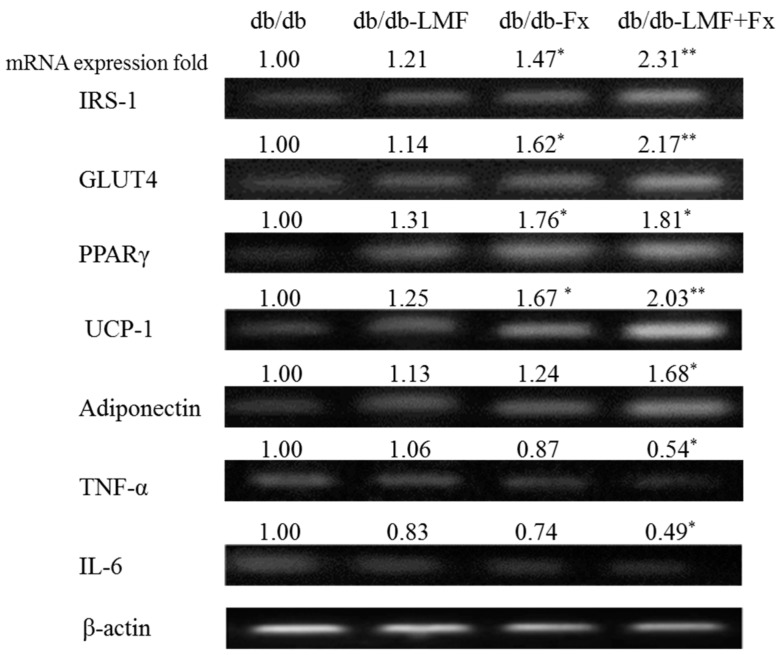
Effects of LMF, Fx, and LMF + Fx on the messenger RNA expression of insulin receptor substrate-1 (IRS-1), glucose transporter type 4 (GLUT4), peroxisome proliferator-activated receptor-γ (PPARγ), uncoupling protein-1 (UCP-1), adiponectin, tumor necrosis factor-α (TNF-α) and interleukin-6 (IL-6) in white adipose tissue of db/db mice for 6 weeks. The relative fold of each mRNA expression as compared to the control group was indicated above the mRNA expression signal. Values were expressed as mean ± SD (*n* = 8). * *p* < 0.05 when compared with the db/db group. ** *p* < 0.01 when compared with the db/db group.

Insulin plays an important role in fat deposition through the regulation of adipose tissue lipolysis, adipogenesis, lipogenesis, and glucose uptake [49]. Glucose transporter type 4 (GLUT4) is essential for insulin-induced glucose uptake, antilipolysis, and lipogenesis, and insulin receptor substrate-1 (IRS-1) phosphorylation is the most important step in the insulin-regulated translocation of GLUT4 in fat cells [50]. People with type II diabetes appear to have low cellular IRS-1 expression in adipose tissues, which is associated with low GLUT4 expression and, therefore, impaired insulin-stimulated glucose transport [51]. As shown in Figure 4, the expression of IRS-1 and GLUT4 mRNAs in the adipose tissue of db/db mice were significantly upregulated by Fx (* *p* < 0.05) and LMF + Fx (** *p* < 0.01), whereas those in the adipose tissue of db/db mice supplemented with LMF exhibited relatively marginal increases, suggesting that Fx plays an important role in the translocation and induction of GLUT4. Notably, no reports published to date have suggested that algal fucoidan, including LMF, can significantly activate overexpression of both IRS-1 and GLUT-4.

In addition, our data indicated that the expression of the transcription factor peroxisome proliferator-activated receptor-γ (PPARγ) mRNA in db/db mice treated with Fx (1.76-fold) and LMF + Fx (1.81-fold) was elevated (Figure 4). PPARγ is the key regulator of adipocyte differentiation and lipid storage promotion [52], and elevated PPARγ has been reported to improve insulin sensitivity [53]. PPARγ has also been shown to increase the expression of mitochondrial uncoupling proteins such as UCP-1 [54]. As shown in Figure 4, the expression of UCP-1 mRNA in db/db mice supplemented with Fx (1.67-fold) and LMF + Fx (2.03-fold) was increased. Previous studies have shown that fucoxanthin significantly modulates diabetes symptoms by inducing UCP-1 expression in white adipose tissues [55], suggesting that fucoxanthin acts as a metabolic regulator of lipids in white adipose tissues. The thermogenic activity of brown adipose tissues is dependent on the level of UCP-1 expression; however, almost all adipose tissues in humans are white adipose tissues [56]. Therefore, UCP-1 expression in white adipose tissues is expected to be an attractive target for the development of antidiabetic therapies.

PPARγ activation has also been shown to inhibit TNF-α expression in the adipose tissue of obese rodents [57]. Our data indicated that the expression of adiponectin, TNF-α, and IL-6 mRNAs was significantly modulated only by LMF + Fx supplementation (Figure 4). Hosokawa et al. indicated [58] that fucoxanthin metabolites reduced IL-6 mRNA expression and prevented the downregulation of nitric oxide synthase mRNA expression by TNF-α. A reduced adiponectin level is a characteristic of type II diabetes [36], and the overexpression of TNF-α and IL-6 induces insulin resistance in adipose tissues [59]. These findings suggested that the combination of LMF and Fx can directly suppress the production of inflammatory adipocytokines. LMF + Fx showed a synergistic improvement on urinary glucose and mRNA expression of IRS-1, GLUT4 and uncoupling protein-1 (UCP-1) in adipose tissue (Figure 3 and Figure 4); as a result, we suggested that the supplementation of LMF and Fx was better than that of Fx or LMF alone. The effects of the LMF, Fx, and LMF + Fx on blood sugar/insulin resistance (Figure 2) might not be significant, but this phenomenon was also observed in other reports. Maeda, et al. [26] showed that supplementation of Fx and normal diet could significantly improve the mRNA expressions of GLUT4, β-adrenergic receptor (Adrb3), and TNF-α in the mice, which had previously been supplemented with a high fat diet for 10 weeks. The mice fed with a normal diet and Fx also had improved blood sugar/insulin resistance, although the reduction of insulin resistance between the mice fed with normal diet and Fx were not statistically different. This might be because insulin signaling is mediated by a complex, highly integrated network, which contains several points of regulation.

## 3. Materials and Methods

### 3.1. Preparation of Low Molecular Weight Fucoidan (LMF) and High Stability Fucoxanthin (Fx)

Fresh *S. hemiphyllum* was collected from the coast of Penghu county, Taiwan over a period of time in December, 2014. LMF was obtained as has been previously described [27]. Briefly, 10 g of dried *S. hemiphyllum* was treated with 500 mL of distilled water and boiled at 100 °C for 30 min. The aqueous solution was separated, lyophilized, precipitated, hydrolyzed by glycolytic enzyme, and then dried to give LMF. The characteristics of LMF were: average molecular weight of 0.8 kDa (87.3%); fucose content 197.4 ± 5.8 μmol/g; sulfate content 32.5% ± 1.9% (*w*/*w*). Purification of fucoxanthin was performed using the method of [60], and Fx was a mixture containing about 10.0% ± 2.3% of fucoxanthin that was coated directly with a polysaccharide film to increase its shelf life and stability. Fx was dissolved in double-distilled H_2_O (ddH_2_O) and completely dissolved with stirring at room temperature for 10 min.

### 3.2. Animal and Diets

Male C57BLKS/J Iar-+*Lepr^db^*/+*Lepr^db^* (db/db) (9 weeks of age) were obtained from Japan Institute for Animal Reproduction (Fukaya, Japan) and were used and maintained under a regular light cycle (12 h light/dark), temperature (22 ± 3 °C), and humidity (50% ± 10%) condition. The db/db mice were fed with a commercial diet (Laboratory Autoclavable Rodent Diet 5010, LabDiet, St. Louis, MO, USA) for 2 weeks after arrival, then the db/db mice were randomly divided into three groups (*n* = 8). During two weeks of acclimation, the blood sugar concentrations of db/db were higher than 240 mg/dL. Thereafter, the control group of db/db mice were treated with saline solution (db/db group), while the other three groups of db/db mice were treated with 300 mg/kg body weight (BW) LMF (db/db-LMF group), 300 mg/kg BW Fx (db/db-Fx group), or 300 mg/kg BW LMF + Fx (a mixture with 50% LMF and 50% Fx, db/db- LMF + Fx group) for 6 weeks. In our preliminary study, LMF [61] and Fx (data not shown) showed no toxicologic effects to mice at 2000 mg/kg BW/day (by using Micronucleus assay); thus, the dosage under 2000 mg/kg BW/day was considered to be safe. LMF/Fx of 100, 300, and 600 mg/kg BW/day were fed to db/db mice for 6 weeks, and the supplementation of 300 and 600 mg/kg BW/day LMF/Fx decreased the blood sugar level. As a result, the dosage of 300 mg/kg BW/day LMF/Fx was chosen in this study. Samples were dissolved into redistilled water to their desired concentration, then mixed and given by gavage to mice every other day for six consecutive weeks. The mice had free access to food and water ad libitum. Food consumption and weight gain were measured daily and weekly, respectively. At the end of the experimental period, the mice were anesthetized with ethyl ether after withholding food for 12 h and urine was taken during this period. Blood samples were taken from the inferior vena cava to determine the plasma biomarkers. Also, the liver and white adipose tissue from inguinal fat were removed after collecting the blood, rinsed with physiological saline solution, and immediately stored at −80 °C until analysis.

### 3.3. Intraperitoneal Glucose Tolerance Analysis

The blood sugar concentration was monitored in the inferior vena cava using a glucometer (Accu-Chek Performa, Roche, Mannheim, Germany). The intraperitoneal glucose tolerance was performed after 6 weeks of treatment, mice were fasted for 12 h, and glucose solution (1 g/kg BW in ddH_2_O) was administered intraperitoneally. Blood sugar concentrations were measured at 0, 30, 60, 90, 120, and 180 min after glucose administration. Areas under the curve (AUC) were calculated using the trapezoidal rule.

### 3.4. Fasting Blood Sugar, Serum Insulin, and Adiponectin Analysis

Fasting blood sugar was measured after 12 h fasting and measured by glucometer. Blood samples were taken from the inferior vena cava. After centrifugation at 1000× *g* for 15 min at 4 °C, the serum was carefully removed from the sample. The concentration of serum insulin was determined using a mouse insulin ELISA kit (Mercodia, Uppsala, Sweden), and serum adiponectin was determined using a two-site enzyme immunoassay (Mercodia, Uppsala, Sweden).

### 3.5. Homeostatic Index of Insulin Resistance (HOMA-IR) and Homeostatic Index of β-Cell Function (HOMA-β)

The homeostatic model assessment (HOMA) is a method used to quantify insulin resistance and β-cell function. It was first described under the name HOMA by [62]. HOMA-IR has been widely used for the estimation of insulin resistance in research, and low HOMA-IR values indicate high insulin sensitivity. It is calculated by using the homeostasis model assessment as follows: 

● HOMA-IR = fasting serum insulin μU/mL × fasting blood sugar mmol/L/22.51

HOMA-β was estimated for β-cell function in research and high HOMA-β values indicate better function of β-cells. It is calculated by using the homeostasis model assessment as follows: 

● HOMA-β = (20 × fasting serum insulin μU/mL)/(fasting blood sugar mmol/L − 3.5) × 100%

### 3.6. Hepatic Enzymes Analysis

Hepatic enzyme source was prepared as previously described by [34]. Briefly, 5 g hepatic tissue was homogenized with 25 mL buffer containing 0.1 M of triethanolamine, 0.02 M of EDTA, and 2 mM of dithiothreitol, pH 7.0. The homogenates were centrifuged at 600× *g* for 10 min to discard any cell debris, and then the supernatant was centrifuged at 10,000× *g* followed by 12,000 × *g* at 4 °C for 20 min to remove the mitochondria pellet. Finally, the supernatant was ultracentrifuged at 100,000× *g* for 60 min at 4 °C to obtain the cytosolic supernatant. Superoxide dismutase (SOD), catalase (CAT), asparate aminotransferase (AST), and alanine aminotransferase (ALT) activity were determined using a commercially available kit (Enzo Life Sciences, Inc., Farmingdale, NY, USA).

### 3.7. Hepatic Glycogen Analysis

One g hepatic tissue was homogenized with 5 mL of a 30% (*w*/*v*) ice-cold KOH solution and dissolved in a boiling water bath (100 °C) for 30 min. The glycogen was precipitated with ethanol, and then pelleted, washed, and resolubilized in distilled water. This solution was treated with an anthrone reagent (0.2% (*v*/*v*) anthrone in H_2_SO_4_), and its absorbance was measured at 620 nm [63].

### 3.8. Urinary Glucose Analysis

The urinary glucose concentration was performed at the last week (sixth week) of treatment, mice were fasted for 12 h and urine was taken during this period. Urinary glucose concentrations were measured by a commercially available kit (Glucose CII-test, Wako, WAKO, Tokyo, Japan).

### 3.9. mRNA Analysis

Total RNA was isolated by RNAzol B (Amersham Pharmacia Biotech, Uppsala, Sweden), and the concentration of total RNA was detected by spectrophotometer (Hitachi, Tokyo, Japan). The synthesis of cDNA was done using Improm-II TM Reverse Transcriptase (Promaga, Madison, AL, USA) according to the manufacturer’s instructions. PCR was performed on the reverse transcribed cDNA product to determine the expression of insulin receptor substrate-1 (IRS-1), glucose transporter type 4 (GLUT4), peroxisome proliferator activated receptor gamma (PPARγ), uncoupling protein-1 (UCP-1), adiponectin, tumor necrosis factor-α (TNF-α), interleukin 6 (IL-6) and β-actin (as an internal control) using a thermal cycler (Biometra, UNO-Thermoboblock, Glasgow, UK). Primer sequences used to amplify the desired cDNA are shown in Table 3. The reactions were carried out in a volume of 25 μL containing 1 unit of *Taq* DNA polymerase (Dong-Sheng Biotech Co., New Taipei City, Taiwan), 0.2 mM dNTP, 10 × reaction buffers, and 100 pmol of sense and antisense primers. After the initial 1 min of 95 °C denaturation, the sense, antisense PCR primers, and amplification sequence protocol were measured and are summarized in Table 1. Above primers were purchased from Mission Biotech Co., Ltd. (Taipei, Taiwan). The PCR products were separated by electrophoresis on 1.2% agarose gels and visualized by ethidium bromide staining under ultraviolet (UV) irradiation. The image of the resulting gel was captured and analyzed by ImageMaster VDS and ImageMaster 1D Elite software (Amersham Pharmacia Biotech, Uppsala, Sweden).

### 3.10. Statistical Analysis

Numerical data are presented as means ± standard deviation. The data was analyzed by a one-way analysis of variance (ANOVA) and followed by the Least Significant Difference test using SPSS (Chicago, IL, USA) version 10 software. *p* < 0.05 was considered a significant difference.

## 4. Conclusions

Our results suggest that LMF and Fx supplementation exerts a beneficial effect on hepatic glucose levels and glucose homeostasis possibly because of the improvement in insulin sensitivity in type II diabetes db/db mice. LMF supplementation was more effective than Fx supplementation on the hepatic glucose metabolism and antioxidant activities in db/db mice, and no significant synergistic effect was observed upon LMF + Fx supplementation. The efficacy of LMF + Fx supplementation on the decrease in urinary sugar and on glucose and lipid metabolism in the white adipose tissue of db/db mice was better than that of Fx or LMF alone, indicating the occurrence of a synergistic effect of LMF and Fx.

## Figures and Tables

**Table 1:** The effect of LMF, Fx, and LMF + Fx for 6 weeks on the level of fasting blood sugar, fasting serum insulin, serum adiponectin, homeostatic model assessment (HOMA) for insulin resistance (HOMA-IR) and β-cell function (HOMA-β) in C57BL/ksj-db/db mice.

Experiments and Indices	Control	LMF	Fx	LMF + Fx
Fasting blood sugar (mg/dL)	475.18 ± 73.04	391.12 ± 47.60 *	392.85 ± 70.27 *	381.56 ± 57.67 *
Serum insulin (μU/mL)	109.95 ± 10.10	99.49 ± 30.22	98.55 ± 19.00	96.82 ± 16.46
Serum adiponectin (μg/mL)	12.25 ± 3.40	24.21 ± 2.87 *	22.34 ± 3.71 *	27.67 ± 1.98 *
HOMA-IR	130.02 ± 26.39	95.59 ± 30.22 *	99.46 ± 34.49 *	93.88 ± 23.48 *
HOMA-β	97.69 ± 14.28	113.30 ± 14.64	107.17 ± 13.15	108.02 ± 20.59

* *p* < 0.05 when compared with db/db group.

**Table 2:** The effect of LMF, Fx, and LMF + Fx for 6 weeks on the levels of hepatic antioxidant enzyme activities, hepatic glycogen, and hepatic function in C57BL/ksj-db/db mice. ALT = alanine aminotransferase AST = aspartate aminotransferase; SOD = superoxide dismutase; CAT = catalase.

Experiments	Control	LMF	Fx	LMF + Fx
Hepatic glycogen (μg/mg liver)	101.34 ± 8.87	120.56 ± 9.90 *	110.25 ± 10.34	118.45 ± 7.43 *
ALT (IU/L)	62.78 ± 6.25	51.56 ± 7.01 *	59.90 ± 6.78	50.89 ± 7.25 *
AST (IU/L)	60.79 ± 2.91	50.05 ± 9.32 *	56.44 ± 3.90	53.60 ± 6.01 *
SOD (U/mg protein)	31.11 ± 4.02	40.84 ± 3.61 *	38.24 ± 3.17 *	39.92 ± 3.08 *
CAT (μmmol/min/mg protein)	2.31 ± 0.71	3.12 ± 0.47 *	3.29 ± 0.39 *	3.34 ± 0.52 *

* *p* < 0.05 when compared with db/db group.

**Table 3:** Primer sequences of mouse mRNA.

Gene Name	Oligonucleotide Sequences	PCR Conditions
IRS-1	F ^1^: AAGGCCAGCACCTTACCTCG	1 min of 60 °C annealing
	R ^2^: AGCCATGGTGGCCCTGGGCAG	1 min of 72 °C extension
GLUT4	F: ACATACCTGACAGGGCAAGG	1 min of 59 °C annealing
	R: CGCCCTTAGTTGGTCAGAAG	1 min of 72 °C extension
PPARγ	F: TGATATCGACCAGCTGAACC	1 min of 58 °C annealing
	R: GTCCTCCAGCTGTTCGCCA	1 min of 72 °C extension
UCP-1	F: TATCATCACCTTCCCGCTG	1 min of 58 °C annealing
	R: GTCATATGTTACCAGCTCTG	1 min of 72 °C extension
Adiponectin	F: GACGTTACTACAACTGAAGAGC	1 min of 57 °C annealing
	R: CATTCTTTTCCTGATACTGGTC	1 min of 72 °C extension
TNF-α	F: GCCTCTTCTCATTCCTGCTTG	1 min of 60 °C annealing
	R: CTGATGAGAGGGAGGCCATT	1 min of 72 °C extension
IL-6	F: ACGGCCTTCCCTACTTCACA	1 min of 65 °C annealing
	R: CATTTCCACGATTTCCCAGA	1 min of 72 °C extension
β-actin	F: GACTACCTCATGAAGATCCT	1 min of 59 °C annealing
	R: CCACATCTGCTGGAAGGTGG	1 min of 72 °C extension

^1^: Forward primer for sequence; ^2^: Reverse primer for sequence.
